# A Molecule-Resolved Bibliometric Analysis of PubMed-Indexed Psychedelic Research (2015–2025): Volume, Temporal Trends, and Publication Types

**DOI:** 10.1192/j.eurpsy.2026.11510

**Published:** 2026-07-31

**Authors:** M. Pastuszak, K. Jakuszkowiak-Wojten, M. Gałuszko-Węgielnik, A. W. Kwaśny, A. Mechlińska, D. Świeczkowski, W. J. Cubała

**Affiliations:** 1 Department of Psychiatry,Faculty of Medicine; 2 Department of Toxicology,Faculty of Pharmacy, Medical University of Gdansk, Gdańsk, Poland

## Abstract

**Introduction:**

Research on psychedelic compounds has grown quickly in the past decade. Yet molecule-specific, quantitative summaries are scattered, making it hard to see how interest differs by compound or over time.

**Objectives:**

To quantify PubMed-indexed publications on ketamine, lysergic acid diethylamide (LSD), N,N-dimethyltryptamine (DMT), psilocybin, and psilocin; to describe annual trends over the last decade; and to identify the most frequently studied compound. Where available, we note planned stratification by publication type (Systematic Review, Scoping Review, Review, Meta-Analysis, Case Report, Classical Article).

**Methods:**

On 28 October 2025 we searched PubMed (MEDLINE) using compound-specific terms and synonyms. Annual counts were extracted from PubMed Timeline and compiled into year-by-year tables. We summarized totals, the share published in 2021–2025, peak annual outputs, and compound annual growth rates (CAGR) for 2015–2025. Publication-type stratification will use the PubMed “Publication Type” field; detailed breakdowns will be presented in figures without inferential analyses.

**Results:**

We identified 9,334 records across five compounds: ketamine 5,704 (61.1%), LSD 1,497 (16.0%), DMT 1,047 (11.2%), psilocybin 999 (10.7%), and psilocin 87 (0.9%). Ketamine led in total and annual volume, with the highest single-year counts (480 in 2024; 475 in 2025). Psilocybin showed the fastest growth, increasing from 12 publications in 2015 to 194 in 2025 (CAGR ≈32.1%) and setting consecutive highs in 2023 (n=148), 2024 (n=187), and 2025 (n=194). DMT rose from 30 (2015) to 112 (2025; CAGR ≈14.1%), peaking in 2024 (n=122). LSD increased from 38 (2015) to 117 (2025; CAGR ≈11.9%) with a 2024 peak (n=122). Psilocin remained small but accelerated from 3 (2015) to 18 (2025; CAGR ≈19.6%), peaking in 2025 (n=18). Concentration in 2021–2025 varied by compound: psilocybin 74.0% (739/999), psilocin 63.2% (55/87), DMT 52.3% (548/1,047), ketamine 38.9% (2,221/5,704), and LSD 34.9% (523/1,497). LSD shows sustained activity since the late 1960s; ketamine has grown steadily over five decades; psilocybin and DMT surged in the 2020s. The most studied compound was ketamine (5,704/9,334; 61.1%). Among serotonergic psychedelics, psilocybin achieved the largest contemporary annual total (n=194 in 2025).

**Conclusions:**

The PubMed-indexed literature on psychedelics is large and still expanding. Ketamine dominates overall output and yearly peaks, while psilocybin is growing fastest, with a new high in 2025. DMT and LSD form substantial secondary literatures—reflecting strong activity since 2020 and long-running interest, respectively—whereas psilocin remains smaller but is accelerating. These quantitative markers, together with forthcoming publication-type profiles, point to a maturing field and a useful baseline for ongoing bibliometric tracking and targeted synthesis.

**Disclosure of Interest:**

M. Pastuszak Grant / Research support from: Beckley Psytech, Compass Pathways, GH Research, MSD, K. Jakuszkowiak-Wojten Grant / Research support from: Alkermes, Allergan, Auspex Pharmaceuticals, Biogen, Cephalon, GH Research, GWPharmaceuticals, Janssen, KCR, Lilly, Lundbeck, Minerva, Orion, Otsuka, and Servier. Maria Gałuszko‐Węgielnik has received research support from: Alkermes, Biogen, Celon, Janssen, GH Research, KCR, Minerva Neurosciences, Lilly, and Servier, Novartis,Compass Pathways, M. Gałuszko-Węgielnik Grant / Research support from: Alkermes, Biogen, Celon, Janssen, GH Research, KCR, Minerva Neurosciences, Lilly, and Servier, Novartis,Compass Pathways, A. Kwaśny Grant / Research support from: Beckley Psytech, Compass Pathways, GH Research, MSD, A. Mechlińska: None Declared, D. Świeczkowski: None Declared, W. Cubała Grant / Research support from: Grants: Acadia, Alkermes, Allergan, Angelini, Auspex Pharmaceuticals, Beckley Psytech, BMS, Celon, Cephalon, Compass Pathways, Cortexyme, Ferrier, Forest Laboratories, GedeonRichter, GH Research, GWPharmaceuticals, HMNC Brain Health, IntraCellular Therapies, Janssen, KCR, Lilly, Lundbeck, MindMed, Minerva, MSD, NIH, Neumora, Novartis, Orion, Otsuka, Recognify Life Sciences, Sanofi, Seaport, Servier Honoraria: Adamed, Angelini, AstraZeneca, BMS, Celon, GH Research, GSK, Janssen, KRKA, Lekam, Lundbeck, Minerva, NeuroCog, Novartis, Orion, Pfizer, Polfa Tarchomin, Sanofi, Servier, Zentiva Advisory boards: Angelini, Celon (ended 2021), Douglas Pharmaceuticals, GH Research, Janssen, MSD, Novartis, Polpharma, Sanofi, Tasman Therapeutics

